# Rapid development of analytical methods for evaluating pandemic vaccines: a COVID-19 perspective

**DOI:** 10.4155/bio-2021-0096

**Published:** 2021-10-14

**Authors:** Jeremy Baldwin, Sakshi Piplani, Isaac G Sakala, Yoshikazu Honda-Okubo, Lei Li, Nikolai Petrovsky

**Affiliations:** ^1^Vaxine Pty Ltd, 11 Walkley Avenue, Adelaide, 5046, Australia; ^2^College of Medicine & Public Health, Flinders University, Adelaide, 5042, Australia

**Keywords:** analytical tools, COVID-19, pandemic, preclinical models, SARS-CoV-2, vaccine design, viruses

## Abstract

Vaccines are key in charting a path out of the COVID-19 pandemic. However, development of new vaccines is highly dependent on availability of analytical methods for their design and evaluation. This paper highlights the challenges presented in having to rapidly develop vaccine analytical tools during an ongoing pandemic, including the need to address progressive virus mutation and adaptation which can render initial assays unreliable or redundant. It also discusses the potential of new computational modeling techniques to model and analyze key viral proteins and their attributes to assist vaccine production and assay design. It then reviews the current range of analytical tools available for COVID-19 vaccine application, ranging from *in vitro* assays for immunogen characterization to assays to measure vaccine responses *in vivo*. Finally, it provides a future perspective for COVID-19 vaccine analytical tools and attempts to predict how the field might evolve over the next 5–10 years.

COVID-19 is a life-threatening disease that emerged in late 2019 that is caused by infection with the severe acute respiratory syndrome coronavirus-2 (SARS-CoV-2). Since its initial detection, SARS-CoV-2 has infected over 200 million people globally and caused almost 5 million deaths [1]. Effective vaccines are critical to help control the pandemic and bring it to an end. Analytical tools and assays are key prerequisites for the design of vaccine immunogens and for assessing the safety, efficacy and durability of vaccine candidates *in vivo*, being critical to each stage of the vaccine development process. When a novel pathogen, such as SARS-CoV-2 emerges, researchers have to start from scratch and rapidly develop new assays, analytical standards and controls in a race against time. Increasing globalization and encroachment of humans on animal habitats means pandemics may become more frequent in the future [2]. Pandemic viruses spread rapidly around the globe and necessitate the acceleration of traditional vaccine development approaches. In the case of COVID-19, vaccine development that typically took up to a decade has been compressed to just a year [3]. For possibly the first time in history, development of analytical tools for vaccine characterization has been conducted alongside, rather in advance of, clinical development, in a process analogous to ‘trying to lay down new tracks in front of a train while it is moving’. Notably, if analytic tools for vaccine assessment are not accurate or reliable, it could lead to delays or, at worst, completely derail pandemic vaccine development efforts.

In addition to trying to better understand the novel SARS-CoV-2 virus, vaccine developers also need to contend with designing or adapting analytical tools to support the evaluation of the new vaccine technologies. The COVID-19 pandemic is unique in that a number of new vaccine platforms, namely viral vectors, mRNA- and DNA-based vaccines, have been deployed to try and counter the virus [4]. While some analytical assays established for traditional vaccines, such as inactivated virus and recombinant protein vaccines, are still applicable to these new classes of vaccines, others are not and require new technology-specific assays and standards to be developed.

This special report first looks at the development process for new analytical tools in the context of an ongoing pandemic in which the disease mechanisms remains largely unknown and the virus presents as a ‘moving target’. It then describes the current state of play of analytical tools been utilized for COVID-19 vaccine development, ranging from optimization of vaccine design to manufacturing and quality assurance, immunogenicity and protection assessment in animals and human trials. The article provides hints, tips and in-house protocols developed by the authors during development of their own SARS-CoV-2 vaccine (Covax-19) and provides insights into emerging analytical tools including bioinformatics and *in silico* modeling that have proved invaluable in their vaccine development efforts.

## The analytical tool development process during an ongoing pandemic

**Figure 1.**
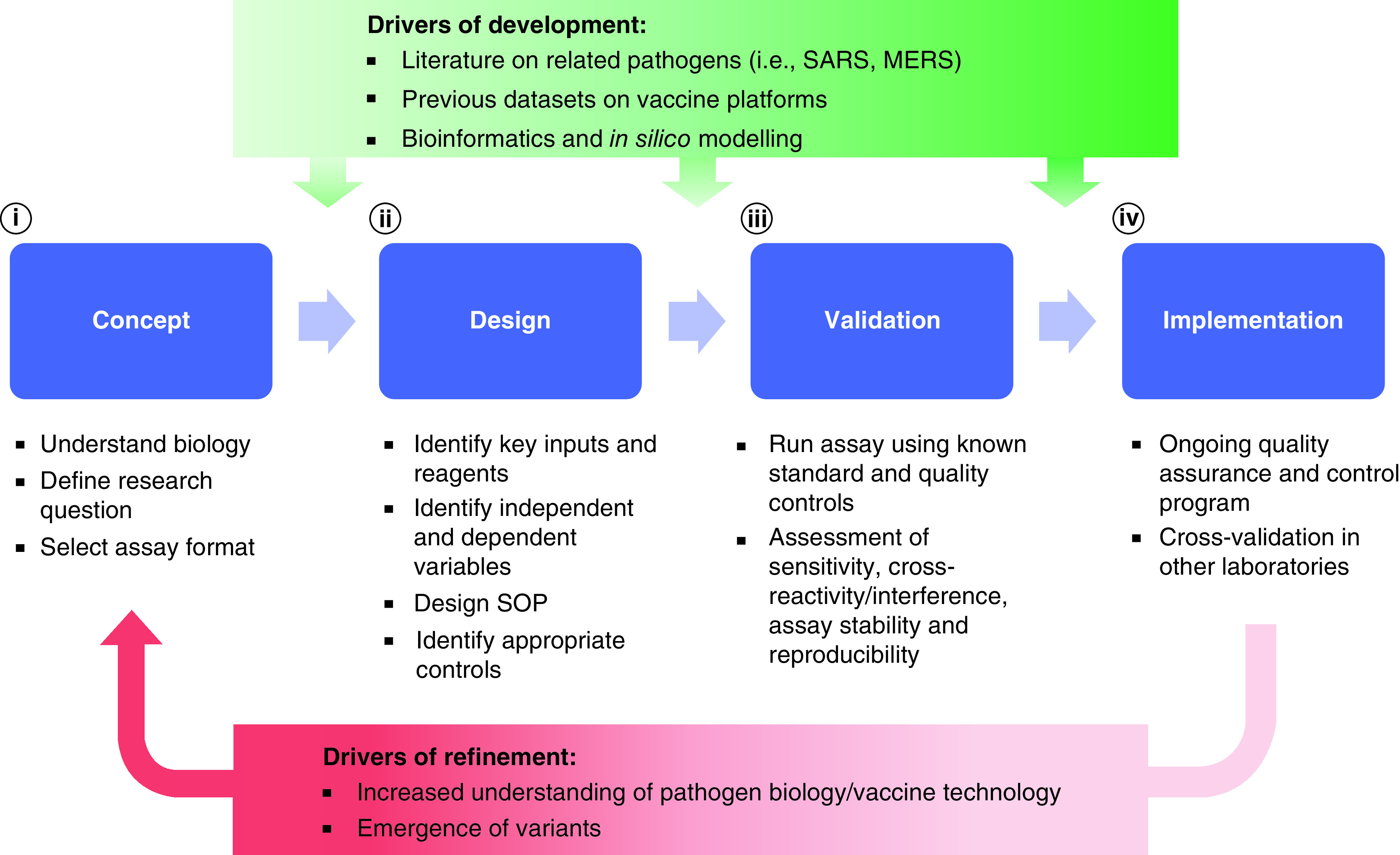
Overview of analytical tool development process in the context of a pandemic. The four main phases of development include **(i)** concept, **(ii)** design, **(iii)** validation and **(iv)** implementation. Factors accelerating the development of analytical tool development are literature on related pathogens, data on vaccine platform and new bioinformatics and *in silico* modeling techniques, while drivers of refinement are increased understanding of virus biology/pathology and emergence of variant strains.

The process of developing analytical tools for vaccine research is similar regardless of whether a researcher is developing an *in vitro* assay or an *in vivo* preclinical model (Figure 1). The first step in developing a new assay is to define the research question that needs to be addressed, thereby allowing selection of the most appropriate analytical method or tool. Next is the design phase, which involves determining assay inputs (i.e., protein, serum, cells), identifying key reagents, development of an assay protocol, assay validation and finally formalization of the protocol into a standard operating procedure (SOP) with specification of required measurements, outputs and interpretation. Validation is critical and once a protocol is established, it needs to be evaluated using known standards and quality controls to assess its sensitivity and specificity, identify potential cross-reactivity/interference with reagents and assess accuracy and reproducibility across the intended purpose. Assay assessment is not static but is a continual process that involves ongoing quality assurance and validation. In a pandemic context, time is critical and as a result assay development needs to be accelerated to keep pace with vaccine development.

### Learning from the past

During the early stages of the pandemic, development of analytical assays can be assisted by literature on previously established assays and biological mechanisms of related viruses. For our SARS-CoV-2 spike protein vaccine development, we adapted techniques and assays used for our previous successful coronavirus vaccine programs against SARS [5] and Middle East respiratory syndrome (MERS) [6] coronaviruses. SARS and MERS have genetic sequence homology of 79 and 50%, respectively, with SARS-CoV-2 and share similar disease pathology [7]. As soon as the reference sequence for SARS-CoV-2 became available in January 2020 [8] we began to adapt assays to assist our development of a SARS-CoV-2 vaccine. This saved valuable time but still considerable adaptation and optimization was needed to adjust these assays to accommodate the new SARS-CoV-2 virus. Notably, not all the previous assays worked for SARS-CoV-2 and hence some new virus-specific assays still had to be developed.

### SARS-CoV-2, a moving target

A key challenge in developing analytical tools is that pandemic viruses continue to mutate and adapt over time, presenting a moving target for both the vaccine and also for analytical assays. At present, there are an average of 200,000 to 500,000 new cases of SARS-CoV-2 confirmed globally each day [1]. Each infected patient contains millions of replicating virus particles under selective pressure, resulting in mutations. These mutations change the virus template and properties, altering the specificity and accuracy of assays. For example, some early polymerase chain reaction (PCR) assays designed to detect the original SARS-CoV-2 reference strain of the virus started to generate false negative results for specific variant viruses that had mutations at the site of the PCR primers [9,10]. In general, analytical processes once developed are fixed, however during a pandemic the assay process needs to be more fluid to accommodate ongoing changes in the pathogen. Open-source initiatives for tracking variants and providing pathogen genome data, such as the National Center for Biotechnology Information (NCBI) SARS-CoV-2 Resources [11], Protein Data Bank Japan (PDBJ) [12] and Nextstrain [13], have helped to ensure assays remain up to date and relevant.

### Accelerating COVID-19 vaccine tool development using *in silico* modeling

The propensity of a pandemic virus to rapidly mutate requires analytical tool development to move at pandemic speed also. Traditional wet lab-based approaches can take months to years to generate data on new viruses and variants. *In silico* modeling is an emerging platform enabling researchers to rapidly model a new virus and its evolving mutations. *In silico* modeling approaches assisted our vaccine program in respect of the design for both the vaccine antigen and suitable analytical assays [14]. The key input for the modeling came with the publication of the SARS-CoV-2 genome sequence on the NCBI database and Protein Data Bank (PDB). Screening the SARS-CoV-genome sequence against the PDB Database, we identified the closely matching SARS spike protein template structure and used this to model the three-dimensional structure of the SARS-CoV-2 spike protein. To help identify the putative cellular receptor for the new virus we performed docking studies of the modeled spike protein against candidate human receptor proteins, thereby identifying angiotensin-converting enzyme 2 (ACE2) as the likely human receptor. This then helped inform our decision-making processes in term of antigen selection, *in vitro* assay designs and choice of pre-clinical animal models. Moreover, as the spike protein continued to mutate and evolve, these new mutations were easily incorporated into our structural models allowing us to predict whether the specificity and selectivity of known SARS-CoV-2 antibodies was likely to be maintained or whether antibody-dependent assays, such as antigen potency assays, ELISAs and neutralization assays, might need to be redesigned.

## Analytical methods for COVID-19 vaccine development

The remainder of this special report covers the analytical assays and tools specific to COVID-19 that have been produced as well as providing future perspectives for the field of pandemic vaccine analytical assays.

## Vaccine characterization

A variety of vaccine technology platforms have been utilized for COVID-19 including mRNA, DNA, viral vector, inactivated virus and subunit protein approaches [4]. The SARS-CoV-2 spike protein has been the primary focus for COVID-19 vaccine design, given the key role played by the coronavirus spike proteins in initiating cell entry [15]. Once a vaccine antigen is selected and synthesized, the first step is to assess its physical characteristics as the properties of the antigen formulation can significantly influence the quality and type of the immune response. Assays are needed to confirm the identity and purity of the vaccine antigen, its structure and chemical composition as well as to detect the presence of harmful contaminants. The various analytical methods for vaccine characterization are detailed below with a critical analysis of the advantages and disadvantages of each approach summarized in Table 1.

**Table 1.** Analytical assays for vaccine characterization.

Analytic tool	Purpose of assay	Advantages	Disadvantages
Polymerase chain reaction (PCR)/reverse transcription polymerase chain reaction (RT-PCR)	Confirm identity of vaccine antigen and vaccine expression systems Detects DNA/RNA of pathogens	– High sensitivity – Rapid results – Equipment (i.e., PCR machine) is readily available in most laboratories	– Susceptible to interference from contamination, in particular carryover from other PCR products – Requires knowledge of target for PCR primer design
Western blot (WB)	Determine identity, size and amount of vaccine using antigen specific antibodies and band size Detect host cell protein (HCP) contamination	– Antigen-specific staining – Size of bands of interest can indicate quality, degradation, post-translation modifications	– Dependent on quality and specificity of antibody – Low throughput – Large amount of protein required for detection – Primarily qualitative/semi-quantitative method – Proteins in denatured form
ELISA	Determine identity and amount of vaccine or detect potential contamination using antigen-specific or pathogen-specific antibodies	– Antigen-specific staining – Low cost – Less time consuming compared with WB – Quantitative method – Protein are not denatured and in native form	– Dependent on quality and specificity of antibody – Harder to detect issues of cross-reactivity of antibodies compared with WB (which can be seen via difference in band sizes) – No information on size of product of degradation
Mass spectrometry (MS) mapping	Determine identity and amount of vaccine. Assess post-modification to vaccine	– Small sample input required – High-throughput and can detect post-translational modifications	– Specialized equipment required which is not widely available0 – Expensive
Fluorescence/UV spectroscopy	Determine tertiary structure of vaccine and correct protein folding	– High sensitivity – Easy method	– Contamination can quench or produce autofluorescence and give false readouts
High-performance liquid chromatography (HPLC)	Determine identity, purity, size, stability and amount of vaccine	– Relative quick – High sensitivity and resolution – Highly automated systems requires minimal training – Can be combined with other techniques such as MS	– Requires high sample input – Expensive
Scanning electron microscope (SEM)	Aggregate formation	– High resolution/ Ultrastructural analysis	– Time consuming – Low throughput – Expensive
Sterility test (direct inoculation/membrane filtration)	Detects presence of microbial contamination	– Low cost – Simple procedure	– Detects only live microbial contamination and provides limited information on identity of contamination
Limulus amebocyte lysate (LAL)	Detects presence of endotoxins in vaccine	– Low cost – Simple procedure	– Animal derived reagents – Batch-to-batch differences
General safety test (abnormal toxicity test)	Toxicity of vaccine/detect adverse events related to vaccine	– Simple procedure	– Ethical concerns related to use of animals – Several studies show poor predictor/correlate of toxicity in humans
High-throughput sequencing	Detect adventitious viruses	– Detect a broad range of viruses (and other pathogens), even if potential source is unknown	– High cost – Requires specialized equipment
Computer modeling (i.e., 3D modeling, molecular dynamic simulations [MDS])	Predict 3D structure and stability vaccine antigen/adjuvant Run docking simulation (cell receptors-virus, virus-antibody, etc.)	– Screen significant number of experimental settings in a fraction of the time as traditional wet-lab based approaches – High predictive power – Reduce costs associated with consumption and labor	– Requires specialized training and computing e-infrastructure

### Identity & purity

Analytical tools for confirming the identity of the antigen vary depending on the type of vaccine. Molecular biology techniques such as PCR, reverse transcription polymerase chain reaction (RT-PCR) and sequencing can be used as critical quality attributes to quickly confirm the identity of mRNA, DNA or viral vector vaccines [16]. These techniques can also be used to validate the identity, copy number and genetic stability of introduced antigen-encoding sequences in the expression systems being used for recombinant protein production. A limitation of PCR-based approaches is that their high sensitivity means they are susceptible to interference from contamination, in particular carryover from other PCR product in the lab [17].

At a proteomic level, there are a number of approaches that can be employed to validate protein-based vaccines, and to confirm the correct translation of mRNA, DNA and viral vectors into protein by host cells. Protein separation using gel electrophoresis followed by staining or western blots can confirm semi-quantitatively protein size and purity. A benefit of this technique is that it is relatively simple procedure with required equipment available in most laboratories. A limitation of western blots is that they require that an antibody is already available against the vaccine protein [18]. More complex techniques such as cryo-electron microscopy, high-performance liquid chromatography (HPLC) and monoclonal antibody surface mapping can provide additional detail on the conformation of the vaccine protein. Studies have shown that SARS-CoV-2 spike protein is not static but undergoes complex conformational changes exposing and hiding potential epitopes [19,20]. For MERS and SARS-CoV-2 locking the spike protein in a pre-fusion conformation has been shown to increase their ability to induce neutralizing antibodies [21,22], indicating protein structure of the vaccine is critical in terms of immunogenicity. In addition, post-translation modifications to the protein such as glycosylation may play an important role in protein stability and immune recognition. The SARS-CoV-2 spike protein is heavily glycosylated and this may shield antibody and T-cell recognition sites [23,24]. The choice of expression systems, for example, use of mammalian or insect cells can influence glycosylation and needs to be characterized [25]. Mass spectrometry techniques are particularly useful to detect post-translational modifications of recombinant proteins and allow these to be compared with the native protein [26]. A limitation of many of these techniques is that they may require specialized equipment and training.

### Vaccine purity & detection of contaminants

For vaccines produced using cell-based expression systems, there is the potential for contamination, such as host cell proteins (HCP), endotoxins and adventitious viruses. As regulatory requirements differ between regions, researchers should consult guidelines and standards released by the relevant regulatory agency such as the US FDA or EMA. Bacterial and fungal contamination can be detected using sterility tests. These tests are routinely performed in all biological manufacturing facilities and generally involve inoculating sterile media with the vaccine sample and the monitoring for 14–28 days for growth via CO_2_ production or visual changes [27]. The assay is straightforward but takes a long time and only confirms the presence or absence of contamination. To determine the exact identity of any bacterial or fungal contaminant requires further time-consuming analysis. Hence newer microbial contamination assays are being developed that can shorten this culture time to 7 days or less, or bypass culture in favor of assays such as PCR that can give same day results. The detection of adventitious viruses in cell substrates can be difficult to detect, even with PCR if the source is unknown, but in recent years high-throughput sequencing assays have been developed to screen for a broad repertoire of different adventitious viruses [28]. Other contamination in the vaccine formulation including microbial byproducts (endotoxin) or chemical residues can be detected by assays such as the limulus amebocyte lysate assay [29] or via a pH change/cell cytotoxicity assays, respectively [30].

New classes of vaccines such as mRNA and DNA are typically chemically synthesized, however several reagents/inputs (i.e., nucleosides, enzymes) are still produced using microbial fermentation and therefore several of the above-mentioned assays are still applicable. Contaminants specific to mRNA vaccines sythensis include oligoribonucleotide impurities as a result of abortive initiation events by RNA polymerases or double-stranded RNA generated by self-complementary 3′ extension, all of which can trigger inflammation [31]. Traditional techniques such as microfluidic capillary electrophoresis were used for assessing the purity and integrity of an mRNA COVID-19 vaccine [32]. In addition, mRNA contamination can be detected using HPLC and double-stranded mRNA-specific monoclonal antibody assays [33].

### Safety

Ensuring safety is the number one priority in vaccine design. A simple test to evaluate the toxicity of biological products is the general safety test (or abnormal toxicity test), which involves administering a defined amount of vaccine to an animal and evaluating subsequent toxicity via body weight loss, clinical scoring, temperature and/or survival [34]. Mice are typically employed for such assays, however being inbred they may have limited predictive ability [35]. In recent years, regulators such as the FDA have dropped the general safety test as a requirement for certain classes of therapeutic products [35,36]. Alternative *in vitro* based assays to measure cytotoxicity, genotoxicity and carcinogenicity in which cell viability, mutations and chromosomal damage is monitored in cells following administration of vaccine components are progressively replacing *in vivo* toxicity testing assays [37].

One early concern for SARS-CoV-2 vaccines was the potential for vaccine-enhanced disease as this had been reported for previous SARS [38,39] and MERS [40] vaccines, as well as dengue [41] and respiratory syncytial virus [42] vaccines. The potential for vaccine-enhanced disease is impossible to assess without access to animal challenge models and this was a major issue for the early SARS-CoV-2 vaccines as such models were not available at that time. Fortunately, vaccine-enhanced disease has so far not been seen to be an issue with any of the SARS-CoV-2 vaccines in clinical use [43].

### Computer modeling

In addition to being used to aid in the development of analytical tools, *in silico* modeling can itself be used as an analytical tool to screen vaccine candidates. 3D structural models of the vaccine immunogen can be used to evaluate the impact of sequence modifications, such as removal or mutation of an enzyme cleavage site or the introduction of stabilizing mutations or the removal of the transmembrane/cytoplasmic domain to create a soluble secreted protein for easy expression and purification. Once the residue changes are incorporated into the spike protein sequence, molecular dynamic simulations (MDSs) can be run to characterize the likely impact of such changes on protein behavior such as whether the modified antigen will form a stable trimer structure similar to the native SARS-CoV-2 spike protein. This computational approach allowed the successful development of our successful vaccine candidate despite our having no access to actual SARS-CoV-2 virus to study [14]. Computational modeling was also used in our laboratory to screen potential adjuvants to boost the vaccine immune response. Synthetic single-stranded oligonucleotides (ODNs) that contain unmethylated CpG motifs mimic bacterial DNA and can bind and activate Toll-like receptor 9 (TLR9) leading to cytokine secretion and enhancement of adaptive immune responses [44]. The traditional approach would involve synthesizing a large library of ODNs and screening them against cell lines, which is time consuming and expensive. A custom program was used to generate 10^16^ synthetic oligonucleotides *in silico* and then a second machine learning program named ‘Search Algorithms for Ligands’ (SAM) was used to select an optimal TLR9 agonist oligonucleotide to include as an adjuvant in our COVID-19 vaccine [45]. While this modeling approach requires access to specialized computing infrastructure, the availability of cloud computing platforms will reduce barriers to such approaches in the future.

### Other vaccine-specific analytical assays

The COVID-19 pandemic is unique in the number of different vaccine platforms that have been deployed against the virus. While some assays established for traditional vaccines may still be applicable to the new types of vaccines such as mRNA or DNA, others are not, requiring new vaccine-platform specific assays and benchmarks to be developed (described in Table 2). A major difference between traditional vaccines and new nucleic acid based technology platforms, such as viral vectors, mRNA and DNA vaccines, is that the vaccines are prodrugs that need to be internalized by host cells in which the nucleic acid is then translated to protein [46,47]. The expressed protein may be secreted by the cell to be seen by B cells or processed internally and presented to T cells by major histocompatibility complex (MHC) molecules on the cell surface. The formulation of carrier (i.e., lipid nanoparticle [LNP]) and expression of viral receptors on host cells used by viral vectors can influence the uptake of the nucleic acid or viral vector vaccines, respectively. The type and location of cell that take up the nucleic acid and express the immunogen may influence the efficacy of the vaccine. There is also the concern that prolonged expression of the immunogen by transfected cells may lead to T-cell killing of the host cells [48]. Yang *et al.* [49] report on analytical method to assess biodistribution of mRNA vaccine in different tissues and organs *in vivo* by incorporating a reporter (i.e., luciferase) into the COVID-19 mRNA vaccine. In combination with follow-up histopathology, this approach can provide a picture of mRNA biodistribution and safety. A limitation of this method is that it requires animal protocol approval, is time consuming, uses specialized imaging equipment and if the reporter is fluorescence-based there may be issues with high auto fluorescent background from host tissue, whereas bioluminescent reporters can vary based on substrate kinetics and tissue uptake [50].

**Table 2.** Vaccine-specific analytical assays for characterization of mRNA and viral vector vaccines.

Analytic tool	Purpose of assay	Advantages	Disadvantages
HPLC/capillary electrophoresis	mRNA purity and contamination	– High resolution/high sensitivity – Quick procedure	– Sensitive to contamination/impurities – Requires specialized equipment – Coelution (two compounds of similar structure pass detector at same time and cannot be distinguished)
Vaccine *in vivo* biodistribution assay	Tracks the uptake of cell internalized vaccines (i.e., mRNA, DNA, viral vector) via reporter	– Provides broad vaccine distribution profile in multiple tissues and organs – Histopathology can provide data on safety and toxicology	– Assay is laborious and time consuming – Autofluorescence of animals (*fluorescent reporter*) and substrate tissue uptake (*bioluminescent reporter*) – Species-specific difference in cell and tissue uptake – High cost – Requires specialized imaging equipment – Requires animal ethics approval
High-throughput allergen sera screening	Binding assays (ELISAs or passive hemagglutination assay) to assess allergenicity potential of vaccine components	– Simple/straightforward procedure – Rapid screening (1–2 h)	– Requires sera from a high number of patients with known allergen history – Limited to IgE-mediated mechanisms
Bioinformatics tool for allergenicity prediction	Analysis tool that uses known allergen databases and algorithms to predict allergenicity of protein sequences	– Rapid screening (5–10 min) – High predictive value, which will continue to increase as reference database increases	– Tools limited to allergenicity prediction of protein-based sequences

As the rollout of vaccines continue, several vaccine platform-specific side effects have been reported, including cases of anaphylaxis and myocarditis after mRNA vaccines (Pfizer and Moderna) [51,52] and ventral venous clotting and thrombocytopenia after adenovirus vector vaccines (Oxford/AstraZeneca and Johnson & Johnson) [53]. New analytical tools might help to pre-screen subjects or refine vaccine formulations to mitigate such side effects. Severe allergic reactions can be triggered by the vaccine antigen itself or one of the other components in the vaccine formulation and can be mediated through IgE and non-IgE-mediated mechanisms [54]. The cause of vaccine-induced allergic reactions and anaphylaxis associated with mRNA COVID19 vaccines has been linked to polyethylene glycol (PEG) in the LNP used in these vaccines, but the exact source remains unclear [55,56]. In general, animal testing has been shown not to be a suitable predicative tool for assessing allergic potential of a compound. A way to pre-screen vaccine components in the future could be to immobilize the vaccine components using binding assay (i.e., ELISAs, passive hemagglutination assay) and then screen sera from patients with documented allergies and/or clinical history of anaphylaxis. The readout is relatively simple and could be adapted for high-throughput screening. The major limitation is the availability of patient sera, with several groups highlighting the need and the utility of an International Sera Bank for use in evaluating the potential human allergenicity of novel compounds and formulations [57]. Alternatively, several groups have utilized bioinformatic approaches to predict allergenicity of vaccine candidates *in silico* [58–60]. Software programs include AllerTOP [61] and AllergenOnline [62]. For viral vector vaccine-induced blood clots, further research is needed, as analytical tool development to predict or avoid such side effects typically first requires a deep understanding of the underlying biology.

## Assessment of vaccine immunogenicity

The two main types of vaccine-induced immune responses are humoral (antibody) and cellular (B and T cell) immunity. Immune assays are required to establish an immune correlate of vaccine protection, that could then be used to guide subsequent vaccine development. A reliable immune correlate of vaccine protection then allows easy comparisons of different candidate vaccines and simplifies future vaccine approvals by avoiding the need to undertake large and expensive Phase III outcome trials. As yet, although there is some correlation seen between serum neutralizing antibody and protection, no immune correlate of protection for SARS-CoV-2 has been agreed [63], making it important for vaccine developers to continue to assess a wide range of immune parameters when testing their vaccine candidates.

### Assays of humoral immunity

Antibody responses to a vaccine are measured mainly via sera collected from venous blood. Unlike cell-based assays, sera can be easily inactivated to allow post infection samples to be safely removed from facilities where virus challenges are undertaken. It is important to run both positive and negative (naive) sera controls in each antibody assay. Examples of available positive sera controls for SARS-CoV-2 antibody assays include the First WHO International Standard for anti-SARS-CoV-2 immunoglobulin, human (NIBSC code: 20/136) and Anti-SARS-CoV-Verification Panel for Serology Assays (NIBSC code: 20/B770). Given the global prevalence of SARS-CoV-2 infection and unprecedented vaccine rollout campaign (as of writing this manuscript there have been over 220 million confirmed infections and 6 billion vaccine doses have been administered globally [1]), the availability of seronegative sera may become increasingly limited, with some groups even using archived sera collected prior to the pandemic to ensure their negative controls are seronegative. In addition, several studies have shown antibody cross-reactivity between SARS-CoV-2 and seasonal human coronavirus antibodies [64,65], with potential to cause background interference in SARS-CoV-2 serology assays.

Quantification of antigen-specific antibodies can be performed using binding assays, such as, traditional ELISA or multiplex bead/protein microarrays. However, neutralization assays are generally more predictive of protection than antibody-binding assays, with the downside that such neutralization assays are cumbersome, expensive and time consuming, have high variability and often need to be performed in high level biosecurity facilities that may not be widely available. Hence as an alternative other simpler functional assays have been developed, such as, virus pseudotyping assays where, for example, a lentivirus backbone is used to express the SARS-CoV-2 spike protein plus a reporter gene, enabling the ability of immune sera to block infectivity of the pseudotype lentivirus to be more conveniently measured. Other functional assays go a step further and completely remove the need to use cells in the neutralization assay, instead measuring the ability of immune sera to block the attachment of the spike protein to the ACE2 receptor bound to an ELISA plate. Each of these assays has its own advantages and disadvantages and a summary of the various analytical assays for measuring vaccine-induced antibody responses is provided in Table 3.

**Table 3.** Analytical assays for measuring vaccine-induced antibody immune response.

Analytic tool	Purpose of assay	Advantages	Disadvantages
ELISA	Antibody quantification	– Easy protocol – Does not require cells	– High background – Low sensitivity
CELISA	Antibody quantification (cell-based)	– Reduce background for certain samples with high nonspecific binding	– Requires cells – Batch-to-batch difference – Difference in protocols between laboratories
Multiplex immunoassays	Antibody detection	– Multiple SARS-CoV-2-specific antibody and other parameters (HLA profile, etc.) can be analyzed in the same sample – High-throughput	– Requires specialized equipment and commercial kits
Capillary electrophoresis	Antibody binding	– Provides data on binding stoichiometry and kinetics of antibodies – Instrumentation required is relatively simple and low cost	– Impurities and solution composition can affect detection sensitivity – Additional sample preparation/purifaction steps may be required
Wild-type neutralization assay	Antibody neutralization activity against native virus	– Mimics natural infection with multiple rounds of entry and replication	– Requires BSL3 facilities not available to many research institutes – Risk that mutations in virus stock occurs during cell culture maintenance
Pseudotype neutralization assay	Antibody neutralization activity against replication-deficient viruses made to express component of the target virus	– Does not require BSL3 facilities – Easily set up in most laboratories around the world	– Allows only a single round of spike-mediated cellular fusion – Virus does not replicate in infected cells – Pseudovirus contains only single component of the virus
Surrogate neutralization assays	Antibody neutralization activity (cell free)	– Easy protocol – Does not require cells or virus – Less variability and batch-to-batch differences – Less expensive – Low biohazard risk	– Does not replicate viral infection – Mode of action limited to binding of spike protein to hACE2 - Only effective for evaluating vaccine that target SARS-CoV-2 spike protein

#### Enzyme-linked immunosorbent antibody assays

ELISA is a technique in which an antigen is either directly coated or immobilized using capture antibodies and then relative levels of specific antibodies can be quantified using a labeled detection antibody. The benefits of ELISA is that it is simple and fast. The assay can be used to detect isotypes/subclasses of antigen-specific antibodies (IgM, IgG1, IgG2a/c, IgG2b, IgG3) and the levels and ratios of these antibodies can provide information on the type of immune response induced by the vaccine. For example, cytokines produced by T helper 1 (Th1) cells and T helper 2 (Th2) cells preferentially bias production of IgG2 and IgG1 antibody subclasses, respectively [66]. A variety of SARS-CoV-2 antibody ELISA assays have reported high background and low sensitivity (<30–40%) particularly at early post immunization time points (7–14 days) [67–71].

#### Cell-based ELISA (CELISA) to measure anti-spike antibody

**Figure 2.**
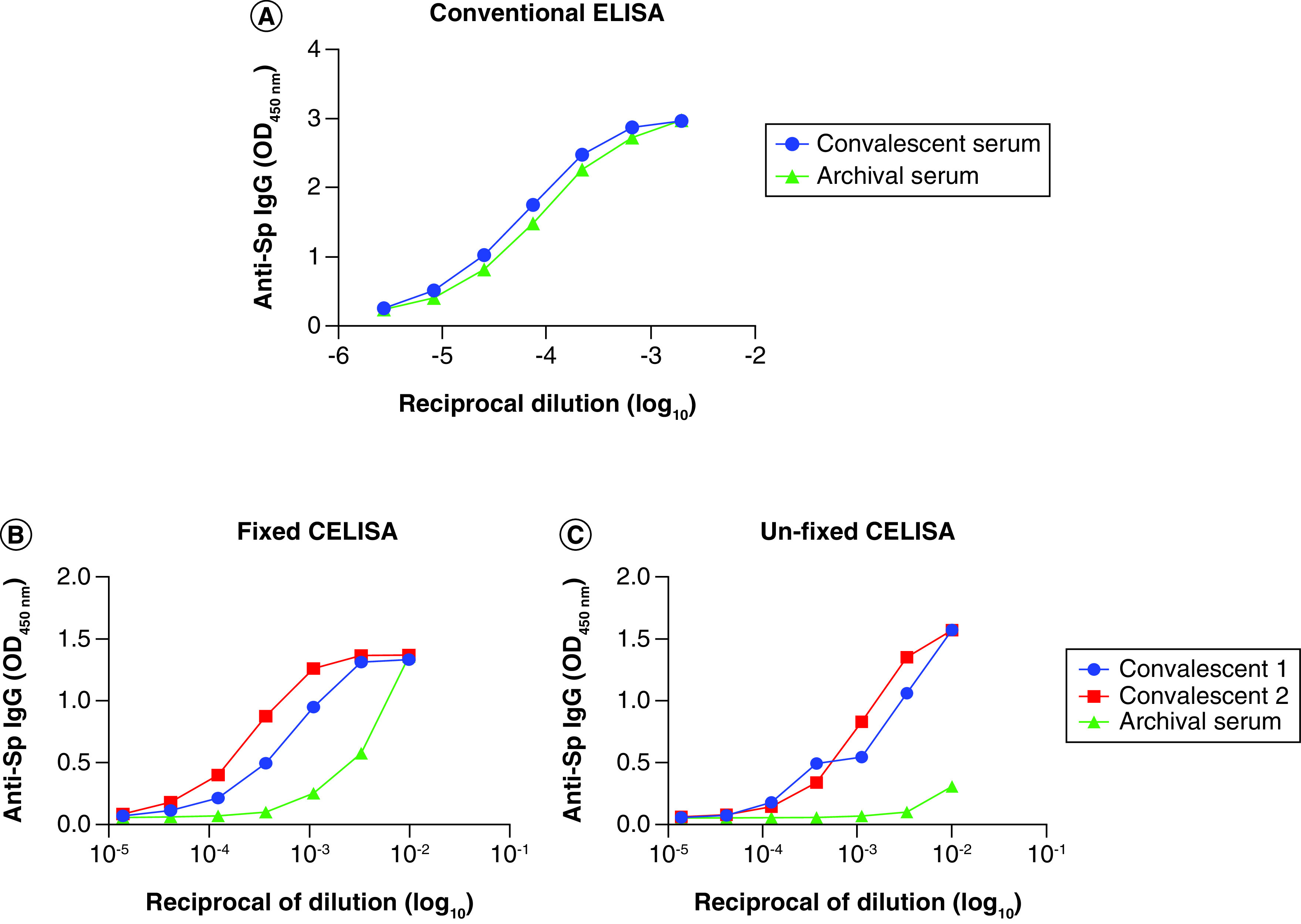
Issues of SARS-CoV-2 ELISA assays. **(A)** Conventional ELISA plates showing high non-specific background with human sera. Cell-based ELISA (CELISA) using either **(B)** fixed or **(C)** unfixed spike-expressing cell clones (HeLa-Sp) using human sera samples showing reduced background.

To overcome the high non-specific binding to sera associated with conventional ELISA with antigen-coated plates, we developed a spike protein cell-based ELISA (CELISA) assay. A full protocol for the CELISA is available in Supplementary Table 1i. In brief, HeLa cells were transduced with a lentiviral vector containing the SARS-CoV-2 spike gene and this then generated stable spike-expressing cell clones (HeLa-Sp). Surface expression of spike protein on the HeLa cells was confirmed by flow cytometry and immunofluorescence staining. An initial cell fixation step was later omitted because it caused a high background. The unfixed CELISA had significantly lower non-specific background when used with human sera compared with the standard spike ELISA (Figure 2). Variations of SARS-CoV-2 Spike protein based CELISA assays reported in literature make use of alternative transfected cell lines (namely HEK293 cells) and analyze samples via flow cytometry, representing antibody binding [72]. Limitations of CELISA assays is batch-to-batch variability common with cell based assays and variation in technical methods and transfection rates making comparison between laboratories difficult.

#### Multiplex immunoassays

A major limitation of traditional ELISA assays are they require high sample volumes and only measure one analyte at a time. Other multiplexed assay platforms including bead-based immunoassays and protein microarrays can be used for detection of antibodies and other analytes in a single sample. For example, the LabScreen COVID PLUS (Lambda) assay measures antibody responses to different fragments of SARS-CoV-2 including full spike extracellular domain, S1, RBD, S2 and nucleocapsid (NP) protein) [73] as well as to MERS-CoV, SARS-CoV and other common seasonal coronaviruses. This helps to identify the potential presence of cross-reactive coronavirus antibodies. Similarly, a chemiluminescent multiplex immunoassay (Vibrant America Clinical Labs) measures antibody subclasses (IgA, IgG and IgM) against different SARS-CoV-2 antigens (S1, RBD, S2 and NP) [74]. These multiplex assays are highly sensitive, require only small serum samples and have the potential to more completely characterize the coronavirus antibody repertoire in infected or vaccinated subjects.

#### Neutralization assays (wild-type vs pseudotype virus)

Neutralization assays are an *in vitro* method to evaluate potential immune protection, although for some viruses such antibody mediated neutralization titers have minimal correlation with actual protection. This is likely because for many viruses T-cell immunity may be at least, if not more, important to virus protection. Hence while neutralization assays provide useful data, they are rarely the sole arbiter of whether a vaccine will be effective or not. A further problem is the difficulty in standardizing such assays as they rely on live cell substrates and these and their culture conditions may vary widely from lab to lab resulting in assay differences. Typically early in a pandemic a panel of convalescent sera is used as a benchmark with the size of the vaccine-induced antibody neutralization responses and allows for comparison to those seen in other laboratories.

The two main types of neutralization assay include wild-type (WT) SARS-CoV-2 and pseudotyped viruses. WT viruses better mimic natural infection, however use of live SARS-CoV-2 viruses requires BSL3 facilities with strict biosafety control, which is not available to many research institutes. In addition, WT viruses can be restricted by local availability of the specific virus isolates needed for the study due to customs importation restrictions. There is also the need to successfully culture and maintain the virus stock with the risk that mutations may occur during cell culture. Pseudotype viruses are replication-deficient viruses made to express component of the target virus, in this case the SARS-CoV-2 spike protein, and these represent a potential alternative to WT virus. The major benefit of pseudotype virus assays is that they only require BSL2 lab conditions, and so can be quick to set up in most laboratories and are flexible to adapt to emerging virus mutants or variants. Our lab developed a lentivirus pseudotype virus assay with fluorescent protein for flow cytometry or imaging-based detection as well as a luciferase-based system to allow a luminescent plate reader detection method (see Supplementary Table 1ii and [14] for further reading). Many other versions of pseudotype virus assays for SARS-CoV-2 have been reported in the literature [75,76].

There may not always be a good correlation between antibody neutralization measured using pseudotype and WT neutralization assays (see correlation graph in Li *et al.* [14]), which may reflect the fact that the two methods measure different virus properties. The WT assay measures the ability to block cell infection by a small pool of viral particles across 3 days of culture whereas the pseudotype assay generally uses a large pool of virus particles as a surrogate for a single spike-driven fusion event. Pseudotype assays allow only a single round of spike-mediated cellular fusion and hence do not mimic a natural infection where there are multiple rounds of entry and replication. It is important to note when interpreting neutralization data that immune protection in patients may involve many different processes including innate immunity, B-cell immunity and T-cell immunity, such that serum neutralization activity is only one facet of this complex system.

#### Surrogate SARS-CoV-2 neutralization assays

Recently, a number of surrogate neutralization assays have been developed to measure antibodies inhibiting SARS-CoV-2 cellular attachment [77–79]. The assay measures the ability of serum antibodies to prevent binding of spike protein to ACE2. Currently two commercial sELISA kits are available: the AdipoGen – SARS-CoV-2 Neutralizing Antibodies Detection Kit, which uses RBD-coated plate and HRP-hACE2, and the GenScript – SARS-CoV-2 Surrogate Virus Neutralization Test (sVNT) Kit, which uses a hACE2-coated plate and HRP-RBD. The major benefit of these surrogate neutralization assays is that they are cell free and faster to run than true neutralization assays, but they suffer from low sensitivity. Their main use is as a quick test to screen plasma samples for potential neutralization under low biosafety conditions.

#### Capillary electrophoresis

Capillary electrophoresis (CE) is another example of an analytical tool that can be used to assess the interactions between viruses and subviral particles (i.e., spike protein) with antibodies and receptors [80]. Viruses and their sub-components can possess charge and be separated according to ionic mobility and size using CE [80]. Analytes separated by CE can then be detected using UV absorbance or with reactive dye or intercalating fluorescent labels and the output data appear as unique peaks based on different migration times. Binding of antibodies to target viral protein alters the migration time and cause a shift in peak of the virus or its components [81]. Altering factors such as molar ratio, pH and buffer solution can provide data on binding stoichiometry and kinetics of antibodies [81]. The benefit of CE is that the instrumentation required is relatively simple and low cost. In addition, CE is a sensitive technique and has even been shown to be able to discriminate between SARS-CoV-2 variants of concern [82]. A limitation of the approach is impurities and buffer solution can affect the detection sensitivity of the assay [83], and the sample (i.e., sera) may require additional preparation or an antibody purification step.

### Cellular immune assays

The primary inputs for analyzing antigenic-specific cellular immune memory responses are splenocytes in animals and peripheral blood mononuclear cells (PBMCs) in humans. Commonly used cellular assays measure antigen-specific B- and T-cell responses by (1) assessing antigen-stimulated immune cell proliferation, ranging from traditional ^3^H-thymidine incorporation assay to carboxyfluorescein succinimidyl ester (CFSE) dilution assay and surrogate proliferation cell markers; (2) cytokine production (i.e., ELISA, CBA, ELISPOT/FLOROSPOT, ICS); (3) assessing the frequency and phenotype of B- and T-cell populations by flow cytometry; and (4) B- and T-cell receptor (BCR and TCR, respectively) repertoire sequencing. Each method has its own advantages and disadvantages and there remains a high degree of variability between laboratories in both technical approaches and interpretation. A summary of the various analytical assays for measuring vaccine-induced cellular responses is provided in Table 4.

**Table 4.** Analytical assays for measuring vaccine-induced cellular immune response.

Analytic tool	Purpose of assay	Advantages	Disadvantages
Proliferation assays	Detection of antigen specific CD4^+^ and CD8^+^ T cells via proliferation	– Direct measure of T-cell division – Compatible with multi-parametric surface and intracellular phenotyping – Single-cell resolution	– High cell input requirements/limit of detection – Limited throughput/scalability
Cytokine production assay (ELISPOT, CBA assay)	Detection of antigen specific CD4^+^ and CD8^+^ T cells via cytokine production	– Low cell input requirement/assay miniaturization – Low limit of detection – Multi-analyte detection/multiplexing – High-throughput scalability – Functional phenotyping	– No surface phenotype – Assay interference (i.e., due to cytokine capture in CBA) – Compatible antibody pair requirements – No direct measurement of cell proliferation
T-cell immunophenotyping	Detection of antigen specific CD4^+^ and CD8^+^ T cells based on surface markers	– Cytokine-independent/Provides information on T-cell activation/memory formation – Single-cell resolution/High sensitivity compared with cytokine production analysis methods – Low cell input	– No information on functionality of T cells
Tetramer assays	Detection of antigen specific CD4^+^ and CD8^+^ T cells via MHC-molecule	– Direct identification/quantification of peptide-specific T-cell populations – Compatible with multi-parametric surface phenotyping – Single-cell resolution	– Assay sensitivity is dependent on T-cell receptor avidity and expression level – Restricted to assessment of predefined T-cell populations based on reagent availability – Limited to donors with specific HLA genotypes
B-cell receptor/T-cell receptor (BCR/TCR) sequencing	Determine antigen recognitions sites and repertoire of CD4^+^ and CD8^+^ T cells	– Useful for evaluating the diversity of an immune response – Can predict binding sites and mode of action against virus and *in silico* method can extrapolate activity against variants/mutations – Can be used to predict off-target effects of vaccines	– Expensive – Data Intensive – Variable amplification efficiency due to differences in GC content, amplification stochasticity, template-switching and polymerase errors – Batch effects due to processing can affect the downstream data analysis

#### T-cell proliferation assays

The proliferation of PBMCs and T-cell subsets (CD4^+^/CD8^+^) following stimulation with antigen-specific stimulation (i.e., live virus, recombinant SARS-CoV-2 spike protein or vaccine antigen) in cell culture setting at different time points can be used to measure the level of T-cell memory and critical for antiviral recall responses [84]. T-cell proliferation can be determined by a number of ways, such as via uptake or persistence of cell-permeant reagents or via surrogate cell cycle markers. Synthetic nucleoside analogue, such as 5-bromo-2′-deoxyuridine (BrdU) [85] and ^3^H-thymidine [86], are taken up and incorporated into the DNA of dividing cells and the proportion of actively proliferating cells in the sample can be quantified via immunochemical or radiographic detection methods. BrdU labelling is more common technique as it is more sensitive and rapid, while ^3^H-thymidine assay is more labor intensive, requires specialized equipment and safety concerns relating to the handling and disposal of radioisotopes. An alternative approach is to assess proliferation via the dilution of cell permeant dyes, such as CFSE, cell trace violet (CTV), or violet proliferation dye 450 (VPD-450). The tracking of lymphocyte division using CFSE is a routine procedure in many laboratories. Prior to antigen stimulation, cells are stained with the cell permeant dyes and as the cells proliferate the intensity of the dye in daughter cells as measured by flow cytometry is halved with each cell division [87]. The only limitation of the assay is that beyond eight daughter cell divisions CSFE fluoresce is too low to distinguish from background autofluorescence and that at high concentrations it can be toxic to lymphocytes. A final way to assess lymphoproliferation is via the expression of surrogate proliferation cell markers, with Ki67 being the predominant marker used in literature [88]. Unlike other assays which continually track proliferation cells, Ki67 only expressed in cells actively proliferating at the time of the assay and therefore only a snapshot at a set time point. Finally, all the proliferation assays, except for radioactive labelling, can be used in conjunction with surface marker (i.e., CD4, CD8, CD62L) to identify the specific subpopulations within the proliferating or non-proliferating cells.

#### Cytokine production assays

The cytokine production profile of immune cells can be used to measure the level and type of immune response induced by vaccine candidates. There are several different methods for analyzing cytokine production; however, the three main assays used to assess immune cell functionality are Cytokine Bead Arrays (CBA), enzyme-linked immune absorbent spot (ELISPOT) and intracellular cytokine staining (ICS) assay. A conventional method for measuring cytokines released by cells is via ELISA, however this technique is very low throughput and requires large sample volume. CBA assays use spectrally distinct beads coated with capture antibodies which allows for the simultaneous measurement of up to 30 secreted analytes from small volumes of biological samples such as supernatant, serum and cell lysate [89]. The concentration of the analyte of the sample can be precisely quantitated allowing to compare results between experiments and laboratories and new enhanced CBA kits capable of detecting sub-picogram concentrations of analytes. The cytometric bead array can be read on any standard flow cytometer equipped with three lasers (i.e., 488 nm/532 nm/633 nm). The major limitation of this technique is that is a bulk measurement of cytokines from a population of cells and cannot provide data on the cytokine-producing cells.

ELISPOT assays allow for the quantitation of the frequency of specific cytokine secreting cells [90]. In brief, immune cells are cultured on membranes coated with cytokine-specific capture antibodies able to bind the secreted cytokines produced by a stimulated cell. After removing the cells via a washing step, cytokine spots are detected via a second enzymatically conjugated cytokine-specific antibody that can produce a colored substrate. The size, intensity and frequency of the spots indicates the number of cytokine-producing cells and level of production. Owing to the high sensitivity and reproducibility of the assay, ELISPOT assays are commonly employed to measure T-cell responses in human clinical trials [91]. Recently ELISPOT analysis on a large cohort of COVID-19 patients demonstrated that although frequency of SARS-CoV-2-specific T cells were similar between asymptomatic and symptomatic patients, asymptomatic individuals showed increased IFN-γ and IL-2 production [92]. However a limitation of the assays is that there is no direct way in an ELISPOT to obtain data on the phenotype of the cytokine-producing cells. In addition, only a few analytes can be measured at a time and the assay cannot detect polyfunctional cells producing multiple cytokines. A new modified technique, known as FLUOROSPOT Assays, in which fluorescent instead of enzymatic conjugates are used for the second cytokine-specific antibody, allows for the detection of multiple cytokines in the same ELISPOT assay [93].

ICS is single-cell based FACS assay that can be used to simultaneously detect both cytokine production and the cell phenotype. Instead of detecting the levels of secreted cytokines, stimulated cells are treated with a selective protein secretion inhibitor, Brefeldin A or monensin, that causes the responding cells to retain cytokines rather than secreting them [94,95]. Brefeldin A inhibits transport between the endoplasmic reticulum and the Golgi while monensin inhibits *trans*-Golgi function [96]. Monensin can be more cytotoxic to certain cell types, whereas Brefeldin A can affect the surface expression of activation markers on T cells [97,98]. Therefore, the choice of inhibitor can depend on the application and research question being addressed. The cells are fixed and permeabilized to allow to detection of intracellular proteins with fluorescently conjugated cytokine-specific antibodies followed by flow cytometry. ICS has been used to detect SARS-CoV-2-reactive CD8^+^ T-cell responses in individuals with COVID-19 [99,100]. Up to 70 and 100% of convalescent individuals showed detectable CD8^+^ and CD4^+^ T-cell responses, respectively, by ICS following *in vitro* stimulation with SARS-CoV-2 overlapping peptides. A limitation of the assay is low sensitivity when frequency of antigen-specific T cells is low and the protocols require that cells are fixed which may interfere with analysis of surface markers.

#### T-cell tetramer assays

Quantifying the number of antigen-specific, MHC-restricted, T cells is important in assessing the cellular immune response generated by potential COVID-19 vaccine candidate. One of the limitations of stimulation-based functional assays, such as proliferation, ELISPOT assays and ICS, is that they only detect functional antigen-specific T cells as determined by the scope and measurement parameters of the assay(s), and therefore can underestimate total antigen-specific T cells. Tetramers can be used to capture the frequency of antigen-specific T cells regardless of functionality, overcoming the limitations of existing stimulation-based functional assays [101]. Tetramers are synthetic structures that are made up of four major histocompatibility complex (MHC) molecules that are linked together and present an antigen-specific peptide (i.e., SARS-CoV-2 antigen) that can bind TCRs specific to the respective cognate peptide [102,103]. The tetramer complexes are labeled with a fluorophore allowing subsequent analysis of tetramer bound T cells via applications such as flow cytometry. Tetramer can be used to detect and isolate rare antigen-specific T-cell populations, such as antigen-specific naive T cells that play an important role in immunodominance [104]. Unlike functional based assays, tetramer staining doesn't require prior stimulation and therefore T-cell population can be segregated into various phenotypic populations without manipulation or distortion of the cells. SARS-CoV-2-specific αβ T cells have been detected using MHC class I multimers and their phenotypic characteristics described across the full spectrum of exposure, infection, and COVID-19 severity and recovery [105,106]. In a recent study, cytokine secretion assays were combined with MHC class I multimer staining to determine the proportion of functional (based on IFN-γ production) and non-functional SARS-CoV-2-specific CD8^+^ T cells [107]. One of the main limitations of tetramers, in particular in the context of a pandemic, is that knowledge of the pathogens epitopes is needed, requiring considerable prior research, when time is a critical factor. In addition, as tetramers are HLA-specific and hence knowledge of a subject's HLA is required to create matching tetramers, limiting broad application in large vaccine clinical trials. New techniques such as high-throughput HLA binding assays and T-cell epitope prediction algorithms are helping reduce assay development times [108].

#### T-cell immunophenotyping

T-cell responses are characterized by activation, clonal expansion/proliferation and differentiation into effector cells to mediate specific functions including memory cell formation [109]. Fluorescent-conjugated antibody staining followed by flow cytometry can be used to phenotype antigen-specific immune cells. The expressions of TCR-dependent activation markers can be used to identify antigen-specific CD4^+^ and CD8^+^ T cells following immunization or re-stimulation *in vitro*. Markers expressed on T cells early after stimulation include CD69 [110], OX40 (CD134)/OX40L (CD252) [111] and CD137 (4-1BB) [112,113] which are upregulated within a matter of hours. Other activation markers expressed later include CD25 which is upregulated by 24 hours and remains elevated for a few days [110,114] and HLA-DR which appears at the late stages of T cell activation [115]. Activation induced marker (AIM) assays (namely CD137^+^ and OX40^+^ for CD4^+^ T cells and CD69^+^ and CD137^+^ for CD8^+^ T cells) have been used in a number of SARS-CoV-2 studies to measure T-cell memory to SARS-CoV-2 for up to 8 months after infection [116] and for determining T-cell response to spike variants [117]. The composition of antigen-specific T-cell populations (naive, memory stem cell, central memory, effector memory and terminally differentiated effector) following vaccination may correlate with vaccine efficacy and duration of protection [118]. Commonly used markers to distinguish T-cell subsets include CD62L, CCCR7, CD45RA and CD45RO [119]. Research on whether a particular T-cell subpopulation correlates with protection against SARS-CoV-2 is still ongoing, but preliminary data of severe versus mild and/or non-severe COVID-19 patients suggests that early establishment of T cell memory predicts a better clinical outcome [120,121].

#### BCR/TCR repertoire sequencing

Antigen-specific B- and T-cell responses are governed by the specificity and selectivity of their respective BCRs or TCRs. BCR and TCR repertoires generated in an immune response can described as (i) skewed, (ii) immunodominant’, (iii) restricted or (iv) limited [122]. Analyzing the full repertoires of BCRs and TCRs provides a better understanding of the immune response. Mathematical predictions by various groups has shown that the number of potential different BCR or TCR sequence combinations is high (up to 10^18^) [123]. The antigen binding site of BCRs and TCRs contain three variable loops, termed complementarity determining regions (CDRs). While CDR1 and 2 are encoded by variable (V) genes, CDR3 is encoded by the segments of the V gene, diversity (D) and joining (J) genes, and displays a high degree of sequence variability [124]. CDR3 also interacts most closely with the antigenic peptide and therefore the diversity of CDR3 amino acid sequences provides a direct measure of diversity of the antigen-specific T/B-cell repertoire [122,125]. High-throughput sequencing (HTS) technology can be used to rapidly decipher the full-length TCR and BCR gene sequences [126,127]. The technique can be used to evaluate the composition of TCR/BCR CDR3 regions of clones (i.e., length, V(D)J segment use, nucleotide insertions/deletions) with sequence-level resolution and provide an overview of the size and diversity of TCR/BCR repertoire in individuals [127]. A recent study showed that a decrease in TCR diversity and a skewed CDR3 length usage in COVID-19 patients with pneumonia compared with those with mild disease suggesting a correlation between TCR repertoire and disease severity [128].

### SARS-CoV-2 animal challenge models

Pandemic vaccine development requires suitable animal models in which to evaluate vaccine efficacy against infection. Although *in vitro* assays can provide useful information on vaccine antigen structure and stability, the best method to determine whether a vaccine is effective is an animal challenge model. Important considerations include the animal species, sex and age and what assays to use to assess protection. Most important is what virus will be used in the challenge and whether it needs to be adapted to ensure it is infectious in the relevant animal species. There are a number of different animal models available to evaluate SARS-CoV-2 infection and each provides different information. Types of models include (i) direct virus challenge studies, (ii) indirect passive immunization studies and (iii) virus transmission models. In direct challenge studies an animal is immunized and then challenged with SARS-CoV-2 virus to assess vaccine protection. Passive immunization studies involve the transfer of isolated antibodies from immunized animal to a naive animal that is subsequently challenged to determine whether the transferred antibodies can confer protection. The benefit of passive immunization is that you can assess the contribution of humoral immunity only in protection. In addition, antibodies can be isolated from clinical trials and transferred to animals to assess whether vaccine-induced human antibodies can confer protection against SARS-CoV-2 infection *in vivo* outside the patient in a controlled experimental setting. Transmission models assess whether the virus infection is transmitted from infected animals to non-infected animals and hence whether vaccination of one animal can prevent it spreading the disease to a second non-vaccinated animal.

The three main methods for assessing protection in animal models are weight-loss, sickness scores and histopathology analysis of tissues of interests (i.e., lung tissue and nasal turbinate). For histopathology analysis, tissue are fixed, sectioned and stained, namely with hematoxylin and eosin (H&E), to reveal tissue morphology. SARS-CoV-2 pathology shares similar characteristics as SARS and MERS infection [129]. Features used in assessment of tissue scoring of SARS-CoV-2, include numbers of lesion and evidence of bronchitis, alveolitis, vasculitis and interstitial inflammation [130–132]. Key factor in the accurate analysis of tissue is correct tissue processing to avoid artifacts that may obscure results and that scoring of tissue is conducted by a certified veterinarian pathologist.

Additional important consideration to take into account in challenge studies is the strain and dose of SARS-CoV-2 virus together with the species, age, weight and co-morbidities of the animals being challenged [133,134]. Furthermore, the timing/duration between last immunization and subsequent viral challenge with SARS-CoV-2 can potentially influence the study results. After immunization antigen-specific IgG antibody titers increase to a peak value usually within 2–4 weeks after a booster immunization, after which time antibody levels progressively decline. Many SARS-CoV-2 challenge studies reported in literature thus far have been performed 1–4 weeks following last immunization [22,135–137] when innate immune system is activated and circulating antigen-specific antibody levels are at their highest, and these early challenge time points may artificially bias vaccine experimental groups and not realistic of the clinical setting.

An ideal animal model should have a similar physiology and closely replicate disease mechanisms/pathology in humans. It is also important that the receptors/pathways are similar in the animal species in order to ensure the animal results replicate the vaccine immune response and virus protection in humans. Due to homology, shared cell entry mechanisms and similar disease pathology of SARS to SARS-CoV-2, several of the previously established SARS animal models in literature were able to be rapidly adapted for SARS-CoV-2 challenges. Animal models established for SARS-CoV-2 include transgenic ACE2 mice, hamsters, ferrets and non-human primates (NHPs) [134]. Mice are used extensive in vaccine research due to their low cost and ease of manipulation, however there is low homology between mouse and human ACE2 (hACE2), limiting their ability to be infected with SARS-CoV-2. For SARS, this issue was overcome through the development of transgenic hACE2 mice [138]. hACE2 mice have similarly been shown to be suitable for SARS-CoV-2 infection [132,139,140]. A limitation is the significant difference between immune systems, physiology and disease pathology of mice and humans [141]. Ferrets, hamsters and monkeys express ACE2 similar to hACE2 endogenously and do not need to be genetically modified [142–144]. In hamsters, SARS-CoV-2 is able to replicate effectively and they display outward symptoms (weight-loss/respiratory distress) [145,146]. In ferrets, viral replication and lung pathology are observed following SARS-CoV-2 infection, but they only exhibit moderate symptoms and no lethality [147]. Macaques show the greatest pathological features to SARS-CoV-2 infection, and most closely match human physiology [148], however high costs and limited availability limit their use.

### SARS-CoV-2 virus quantification

Tissue for SARS-CoV-2 virus quantification can come in many formats including blood sera, tissue specimens and mucosal fluids. The sensitivities and detection rates for SARS-CoV-2 can vary depending on location and timing of specimen collection [149–151]. Moreover, the method used to obtain the samples may influence the results, for example, nasal wash can collect high amounts of extracellular virus while nasal swab through mechanical disruption can collect more infected cells epithelial cells from mucous membrane and therefore provide additional data on intracellular SARS-CoV-2.

Virus quantification can be performed using plaque-based assays, tissue culture infectious dose (TCID50) assays [152], PCR assays [152] or *in situ* tissue staining [153]. Plaque assays determine the number of plaque forming units (pfu) of virus on a confluent monolayer of cells, while TCID50 is an end point dilution assay determines the virus dose resulting in a cytopathic effect in 50% of inoculated tissue culture cells [154]. Both the plaque and TCID50 assays represent the gold standard in quantifying live SARS-CoV-2 virus. The PCR method quantifies SARS-CoV-2 genetic material in a sample and uses this to extrapolate the amount of virus. The benefit of the PCR approach is that it is easier to perform and can be conducted on inactivated samples, but it detects both active and inactive virus and thus has a higher background. Furthermore SARS-CoV-2 virus variants may be missed by the PCR due to mismatch between primers and new genetic sequence template [155]. *In situ* staining using immunohistochemistry (IHC) or *in situ* hybridization (ISH) is another way to quantify virus infection [153]. IHC involves the use of antibodies to detect viral proteins while ISH involves the use of nucleic acids probes to localize a specific DNA or RNA sequence [156]. These techniques provide spatial distribution and information on the type of cells that are infected, which may be important in understanding and interpreting disease pathology. Important factors affecting staining is processing of the tissue such as tissue fixation, antigen retrieval method and inclusion of positive control slides [156].

## Conclusion

A vaccine is only as good as the analytical tools available to evaluate it. In the event of a pandemic, such as COVID-19, there is added pressure to rapidly develop analytical tools to help bring safe and effective vaccine candidates to market in a timely manner. In this special report, we have detailed the analytical tool development process in the context of a pandemic vaccine and the common pitfalls faced by vaccine researchers and developers. Emerging technologies, such as computational modelling and bioinformatics, are helping overcome these challenges and changing the way we design and validate analytical methods and assess the vaccines candidates themselves. In this Special Report we have described the current state of analytical methods relevant to COVID-19 vaccine ranging from antigen characterization to *in vivo* protection models. Other critical factors in rapid deployment of analytical tools during pandemics are open-source protocols, liberal sharing of appropriate controls/standards to allow benchmarking of results between laboratories, and rapid dissemination and publication of ongoing research into biology and disease mechanisms of the pandemic virus to ensure all analytical tools and vaccine designs remain accurate and relevant.

## Future perspective

COVID-19 is not the first global pandemic in modern history and will not be the last. Increased globalization and urbanization/human-animal contact mean that the frequency and severity of future pandemics are likely to increase [2]. Vaccines are key to responding to pandemics; however, robust analytical tools are needed to design and evaluate potential vaccine candidates. Lack of suitable assays can slow the vaccine development process and leave vulnerable populations unprotected. There has been a significant media attention on successful COVID-19 candidates, with less attention given to equal number of failed vaccine candidates, such as a recombinant vesicular stomatitis virus vaccine (V590, IAVI-Merck), a measles virus vector vaccine (V591, Merck) and a protein subunit vaccine using a HIV-derived trimerization domain (V451, UQ-CSL). Closer attention to vaccine analytical assay design during the early development of such vaccines may have prevented these failures and saved significant costs. Hence, in order to better tackle future pandemic threats we need to improve analytical tools for rapid vaccine development.

Key analytical trends that are likely to influence the pandemic vaccine field over the next 5–10 years include:

- Modular design: Given the increase in novel viruses and variants it is important to design modular analytical tools that are adaptable and can be readily validated so they can be rapidly deployed.
- Multiplex assay formats: An individual analytical assay can provide single output/measurements; however, by integrating the results and connecting datasets from different assays enable researchers to identify new trends, biomarkers, correlates of protection and vaccine antigen targets
- Integration of emerging technologies: Throughout the paper we highlight the benefit of incorporating new technology platforms, such as bioinformatics and *in silico* modeling, in speeding up the design and validation of vaccine antigen and analytical assays to keep pace with our changing world. Other emerging technologies such as next generation bulk and single cell RNA-sequencing can be used to broadly characterize the entire expression profile of immune cells, this approach is particular important in the context of a novel virus wherein correlates of protection are unknown and/or when new adjuvants or vaccine technologies are being employed and their mode of action has yet to be fully characterized. However a limitation of these technology platforms is the significant amount of technical knowledge and infrastructure required to run analyses. Training in these new areas of research, even starting at an undergraduate curriculum level, and increasing the availability and access to computing platforms and other specialized equipment will help with the uptake of these technologies.

**Executive summary**

- Key in pandemic vaccine analytical tool development is to tightly frame the research question that needs to be addressed, identify key inputs and design a reproducible protocol with appropriate controls to allow assessment of assay sensitivity, specificity, accuracy, reproducibility and interference.
- Developing analytical tools is not a fixed process but should be ongoing with the method being continually checked/validated and updated if necessary.
- In silico modeling tools can help speed up pandemic vaccine development by enabling researchers to rapidly design and evaluate vaccine candidates.
- Robust characterization of the antigen and continued quality assurance measures are necessary as the composition of the final vaccine product can influence its immunogenicity and protection.
- Newer functional antibody assays such as pseudotype and surrogate neutralization assays help overcome limitations of conventional virus neutralization assays.
- Analytical tools for use in development of pandemic countermeasures should ideally be adaptable to respond to changing circumstances including rapidly mutating viruses.
- Early pandemic vaccine development is hindered by lack of access to appropriate immune sera and antigen standards and creation and distribution of such standards should be an immediate priority in event of any outbreak.
