# Supplementary material for: Rapid development of analytical methods for evaluating pandemic vaccines: a COVID-19 perspective
Source: Bioanalysis. 2021 Oct 14:10.4155/bio-2021-0096. doi: 10.4155/bio-2021-0096 (PMC8516068; doi:10.4155/bio-2021-0096)
Supplement: Supplementary file 1 [file Supplementary_Table_1.docx]

**Supplementary table 1:**

| **IN-HOUSE PROTOCOLS DEVELOPED FOR SAR-COV-2** |
| --- |
| **(i) CELL-BASED ELISA (CELISA)** |
| Spike coding sequence of SARS-CoV-2 was synthesized at GenScript and cloned into pCDH-EF1-MCS-IRES-Puro vector (System Biosciences). Lentiviral particles were generated using 293TN cells (System Biosciences) using psPAX2 (Addgene) and pMD2.G (Addgene) packaging plasmids. Early passage HeLa cells were infected with SARS-CoV-2 Sp lentivirus and selected with 2 µg/ml of puromycin 48 h after infection to generate stable cell clones (HeLa-Sp). Surface expression of Sp was then confirmed by flowcytometry and immunofluorescence staining. For CELISA assay, 1.5 x 10^4^ HeLa-Sp cell were transferred in a volume of with 100 μl of DMEM+5% FCS into each well of the microtitre plate (Greiner Bio-One, Cat. No. 655180) then incubated overnight at 37°C in 5% CO_2_. For fixed CELISA after removing culture supernatant, plate was washed with PBS 3 times and 4% formaldehyde for 20 minutes at RT. For un-fixed CELISA, cells were washed with DMEM containing 5% FCS. After washing with 300 μl/well 3 times, 100µl diluted sample (sera) were added and incubated 1 h at 37°C in 5% CO_2_. After washing 3 times, biotinylated species-specific IgG or IgM antibodies (Invitrogen) with HRP-conjugated Streptavidin (BD Biosciences) for 1 h at 37°C in 5% CO_2_. After washing, TMB substrate (KPL,SeraCare, Gaithersburg, MD, USA) was added and incubated for 10 min before the reaction was stopped with 100µl 1M Phosphoric Acid. The optical density was measured at 450nm (OD450nm) using a VersaMax plate reader and analyzed using SoftMax Pro Software. Average OD450nm values obtained from negative control wells were subtracted. |
| **(ii) SARS-CoV-2 Spike Pseudotyped Neutralization Assay** |
| A non-replicative SARS-COV2 Spike pseudotyped lentivirus-based platform was developed to evaluate neutralization activity in infected/convalescent sera in a BSL2 facility. The hACE2 ORF (Addgene# 1786) was cloned into a 3rd generation lentiviral expression vector pRRLSIN.cPPT.PGK-GFP.WPRE (Addgene# 122053) and clonal Hek 293T cells stably expressing ACE2 were generated by lentiviral transductions as described previously (PMID: 22685410), followed by single cell sorting into 50% Hek 293T conditioned media (media conditioned from 50% confluent Hek 293T cultures). Lentiviral particles pseudotyped with SARS-COV2 Spike envelope were produced by co-transfecting Hek 293T cells with a GFP encoding 3rd generation lentiviral plasmid HRSIN-CSGW (a gift from Camille Frecha: PMID 15029233), psPAX2 and plasmid expressing codon optimized but C-terminal truncated SARS COV2 S protein (pCG1-SARS-2-S Delta18, herein Spike Delta18). Neutralization activity of donor sera was measured using a single round transduction of ACE2-Hek293T with Spike pseudotyped lentiviral particles. Briefly, virus particles were pre-incubated with serially diluted donor sera for 1 h at 37°C. Virus-serum was then added onto ACE2-Hek 293T cells seeded at 2,500 cells per well in a 384-well tissue culture plate a day before. Following spinoculation at 1200xg for 1 h at 18°C, the cells were moved to 37°C for a further 72 hours. Entry of pseudotyped particles was assessed by imaging GFP positive cells and total cell numbers imaged through live nuclei counter staining using NucBlue (Invitrogen). Total cell counts and % GFP positive cells were acquired using InCell imaging platform followed by enumeration with InCarta high content image analysis software (Cytiva). Neutralization was measured by reduction in % GFP expression relative to control group infected with the virus particles without any serum treatment. |
